# The effects of *Salvia przewalskii* total phenolic acid extract on immune complex glomerulonephritis

**DOI:** 10.1080/13880209.2017.1383486

**Published:** 2017-10-12

**Authors:** Yang Yang, Zhi-Peng Wang, Shou-Hong Gao, Hong-Qi Ren, Ren-Qian Zhong, Wan-Sheng Chen

**Affiliations:** a Department of Laboratory Diagnostics, Changzheng Hospital, Second Military Medical University of CPLA, Shanghai, China;; b Department of Pharmacy, Changzheng Hospital, Second Military Medical University of CPLA, Shanghai, China;; c Affiliated Huaihai Hospital of Xuzhou Medical University (The 97th Hospital of CPLA), Xuzhou, China

**Keywords:** *Salvia przewalskii* total phenolic acid extract, rosmarinic acid, salvianolic acid B, proteinuria, therapeutic efficacy

## Abstract

**Context:***Salvia przewalskii* Maxim. (Lamiaceae) is a Chinese herbal medicine that has long been used for the treatment of cardiovascular disease.

**Objective:** The study investigated the therapeutic efficacy of *S. przewalskii* total phenolic acid extract (SPE) on immune complex glomerulonephritis (ICG) in rats.

**Materials and methods:** Sixty-two Wistar rats were randomized into six groups. ICG was induced in all groups except normal control group. SPE was administered intragastrically at 24 h intervals for 40 consecutive days. Urine protein (UP), total serum protein (TSP), serum albumin (SA), serum cholesterol (SC) and serum urea nitrogen (SUN) were measured one day before, on day 20 and 40 after SPE administration. On day 40 after SPE administration, the kidneys were removed and prepared into pathologic sections. In addition, kidney wet mass was measured for calculating the kidney wet mass coefficient (KWMC).

**Results:** UP excretion was reduced significantly on day 20 after SPE administration in all three SPE groups as compared with that in medium group, and this effect was observable continuously until 40 days after SPE administration. Compared with medium group, TSP and SA were increased in all three SPE groups after 40 days treatment, while SC and SUN were decreased. KWMC was decreased significantly in 100 mg/kg SPE group after 40 days treatment compared with that in medium group. Histopathologic analyses showed that renal inflammatory infiltration and kidney intumesce were alleviated in all three SPE groups.

**Conclusions**: SPE may be a potential therapeutic drug for glomerulonephritis.

## Introduction

*Salvia* (Lamiaceae) is an important plant genus consisting of about 1000 species around the world (Huang and Sun 1977). More than 40 species of *Salvia* have been utilized as medical remedies in China (Li et al. 2008). *Salvia przewalskii* Maxim., called *Ganxishuweicao* in Chinese, is a herbaceous perennid. It mainly grows in Gansu, Sichuan, Yunnan and Tibet provinces of China, and has long been used as a traditional Chinese herbal remedy for the treatment of cardiovascular diseases by local inhabitants. The main chemical components of *S. przewalskii* are volatile oil (Liu et al. 2006), diterpenoids (Yang et al. 2008; Ohsaki et al. 2011; Jiang et al. 2013; Xue et al. 2014; Wang et al. 2015), triterpenoids (Wang et al. 1988; Yang et al. 2008), polyphenols, phenolic acids, protocatechuic acids and salvianolic acids (Lu et al. 1991; Wu et al. 1999; Qing et al. 2004; Xue et al. 2014). The pharmacological activities of *S. przewalskii* are similar to those of *Salvia miltiorrhiza* Bunge because of the similar chemical constituents of tanshinones and phenolics (Skała and Wysokińska 2005). *S. przewalskii* is used as a substitute for *S. miltiorrhiza*.

In our previous preliminary study on *S. przewalskii* total phenolic acid extract (SPE), we identified five new diterpenoids and one new monoterpenoid glycoside from SPE, finding that SPE could reduce whole blood viscosity in Wistar rats and increase the urine excretion of water load Wistar rats (Chen et al. 2003; Yang et al. 2011, 2012, 2013, 2017). In addition, we found that SPE could reduce proteinuria and preserve the morphology and structure of podocytes by retaining the level of slit diaphragm proteins in a rat model of puromycin aminonucleoside-induced podocyte injury (Dai et al. 2015).

The aim of this study was to explore the efficacy of SPE more comprehensively in a rat model of immune complex glomerulonephritis (ICG) induced by bovine serum albumin (BSA) and complete Freund’s adjuvant (CFA) in an attempt to explain the underlying mechanism, knowing that this animal model is closely relevant to human mesangial proliferative glomerulonephritis (MsPGN) (Fujita et al. 1991; Jia and Zou 1996).

## Materials and methods

### Plant material

The roots and rhizomes of *S. przewalskii* were collected from Wen County, Gansu Province of China in September 2009 and identified by Prof. ZHANG Hanming from Department of Pharmacognosy, School of Pharmacy, the Second Military Medical University (SMMU) of CPLA (Shanghai, China). A voucher specimen (Batch No. 200909-b15) was deposited in the herbarium of the said School of Pharmacy.

### Preparation of SPE

Fresh roots and rhizomes of *S. przewalskii* were cautiously dried at room temperature of 25 ± 2 °C for three days, and then the raw medicinal material (5 kg) was cut and grounded to powder mechanically in a mill. The dried powder was macerated and percolated with 50 L 50% ethanol solution for 48 h. The solvent was evaporated at 60 °C under reduced pressure to yield dried ethanol-soluble-extracts (0.6 kg). Then, the ethanol-soluble-extracts were chromatographed over the macroporous adsorptive resin AB-8 (0.8–1.2 mm) (Zhentiancheng Technology Co., Ltd., Tianjin, China) eluting with gradient mixtures of water and ethanol. The eluting solution of 50% ethanol was concentrated and turned to be SPE (90 g) (Yang et al. 2012).

SPE was dissolved in double-distilled water containing 0.8% sodium carboxymethyl cellulose (CMC-Na) and left at room temperature for 20 min to infuse. SPE solution was diluted at 5, 10 and 20 mg/mL with double-distilled water containing 0.8% CMC-Na. Then, 1 mL/kg (body mass) of each solution was administered orally to the rats (equivalent doses at 50, 100 and 200 mg/kg with respect to the mass of SPE). These solutions were freshly prepared just before administration.

### HPLC analysis of SPE

The content of two main chemical constituents of SPE, rosmarinic acid and salvianolic acid B, was analysed by high performance liquid chromatography (HPLC). A methanol stock solution of rosmarinic acid and salvianolic acid B was prepared and diluted to proper concentrations to set up the calibration curves. An Agilent 1200 HPLC system (Agilent Technologies, Palo Alto, CA) comprising a quaternary solvent delivery system, an on-line degasser, an autosampler, a column temperature controller and photodiode array detector was used. All data were acquired and analysed in Agilent chemstation software. The chromatographic column was a Dikma Diamonsil RP C_18_ column (5 μm, 250 mm ×4.6 mm). The injection volume was 20 μL and the flow rate was 1.0 mL/min. The column temperature was set and maintained at 30 °C and the detection wavelength was set at 288 nm. The mobile phase consisted of A (acetonitrile) and B (0.05% phosphoric acid aqueous solution). Gradient variations are shown in Table 1.

**Table 1.** Gradient composition of the mobile phase for HPLC analysis of SPE.

Time (min)	A (%)	B (%)
0–20	12 → 20	88 → 80
20–60	20 → 30	80 → 70
60–65	30 → 12	70 → 88

### Reagents and chemicals

Tripterygium glycoside (TG) tablets (Batch No. 040901; Fudan Fuhua Pharmaceutical Co., Ltd., Shanghai, China) were dissolved in 0.8% CMC-Na double-distilled water to get a 1.5 mg/mL solution as the control medicine just before administration. BSA (Batch No. 0409A09; Shenggong Bioengineering Co., Ltd., Shanghai, China) was dissolved in sterilized 0.15 mol/L NaCl solution to 3 mg/mL. CFA (Batch No. 122K8927) was purchased from Sigma Corporation (St. Louis, MO).

### Animals and drug administration

A total of 62 Wistar rats of SPF grade (Slaccas Experimental Animal Co., Ltd., Shanghai, China; Certificate: SCXK2003-0003) weighing 120 ± 15 g were housed in the experimental animal center of the SMMU (Certificate: SYXK2002-0026). The rats were placed in the animal facility at 25 ± 2 °C, 50 ± 2% humidity and 12 h light/dark cycle with free access to food and water for at least one week before the experiment. Rats were randomized into six groups: normal control (N) group (*n* = 12); medium (M) group (*n* = 10); TG control medicine group receiving intragastrical administration of 15 mg/kg TG (*n* = 10); and three SPE treatment groups receiving intragastrical administration of low (50 mg/kg), medium (100 mg/kg) and high (200 mg/kg) dose SPE as SPE1, SPE2 and SPE3 groups (*n* = 10 each). Then, the animals were housed individually in metabolic cages to obtain their urine.

All experimental procedures were carried out according to the protocols approved by the Animal Care Committee of the Animal Center at the Chinese Academy of Sciences, and performed in accordance with the recommendations and policies of the SMMU for the protection of animals used for experimental and other scientific purposes.

### Experimental protocol and treatment

ICG was induced as previously described (Jia and Zou 1996). Briefly, except for N group, one kidney was resected from each of the animals in the other five groups after 10% chloralization before initiation of the experiment (Cheng et al. 1995). BAS (3 mg) and CFA (0.1 mL) were injected in rats at a foot pad on day 1, and again at the end of the first week. At the end of the second week, the animals received a series of intraperitoneal (i.p.) injections of 0.5, 1, 1.5 and 3 mg BSA at 1 h intervals. During the third week, 2 mg BSA was injected i.p. on day 1, followed by 3 mg BSA on day 3, 5 and 7. Then, 100 μg lipopolysaccharide (LPS) of *Escherichia coli* was injected i.p.

SPE and TG were administered intragastrically (i.g.) at the designated doses in 24 h intervals for 40 consecutive days. Urine protein (UP) and blood biochemical indexes were measured one day before, on day 20 and 40 after drug administration. On day 40 after SPE administration, the animals were sacrificed to obtain the kidneys for the preparation of pathologic sections. In addition, kidney wet mass was measured. The kidney histopathologic sections were observed under a light microscope. UP was quantified by the biuret method. TSP, SA, SC and SUN were detected by routine laboratory tests.

### Assessment of kidney function

Measurement of total serum protein (TSP), serum albumin (SA), serum cholesterol (SC) and serum urea nitrogen (SUN) was carried out at three time points, one day before, on day 20 and 40 after drug administration. The kidney specimens were weighed to calculate the ratio of kidney wet mass and rat body mass, which is known as kidney wet mass coefficient (KWMC).

### Histopathological examination

Kidney specimens were examined by light microscopy to evaluate the severity and extent of glomerular lesions. Briefly, the renal tissues were fixed in 10% formalin solution, paraffin embedded, sliced to 4 μm sections, stained with haematoxylin–eosin (HE) and periodic acid Schiff, and finally observed under a microscope by a pathologist who was blind to the experimental protocol.

### Statistical analysis

All results were expressed as mean ± standard deviation, except for histopathological examinations. Statistical analysis was performed using one-way analysis of variance followed by Fisher’s LSD multiple comparison test (SPSS software Version 16.0, SPSS Inc., Chicago, IL). Values of *p* < 0.05 were considered statistically significant.

## Results

### HPLC analysis of SPE

**Figure 1.**
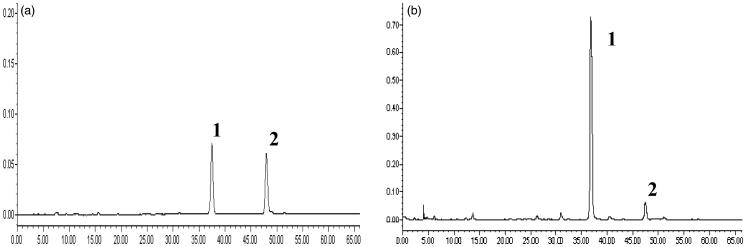
HPLC plots of standard solution (a) and SPE (b). The peaks are identified as: rosmarinic acid (1), salvianolic acid B (2).

The retention time of rosmarinic acid and salvianolic acid B was 48.554 min and 37.264 min, respectively. The calibration curves of rosmarinic acid and salvianolic acid B were obtained by the ratio of a series of peaks area (*Y*) to corresponding concentrations (*X*, μg/mL), and were used to calculate their content in SPE. The content of rosmarinic acid and salvianolic acid B in SPE was 31.58 and 5.52%, respectively. The results of HPLC analysis are shown in Figure 1.

### The effect of SPE on UP excretion

UP excretion in all groups of model animals was significantly higher than that in M group one day before drug administration, indicating that the animal model was established successfully.

**Figure 2.**
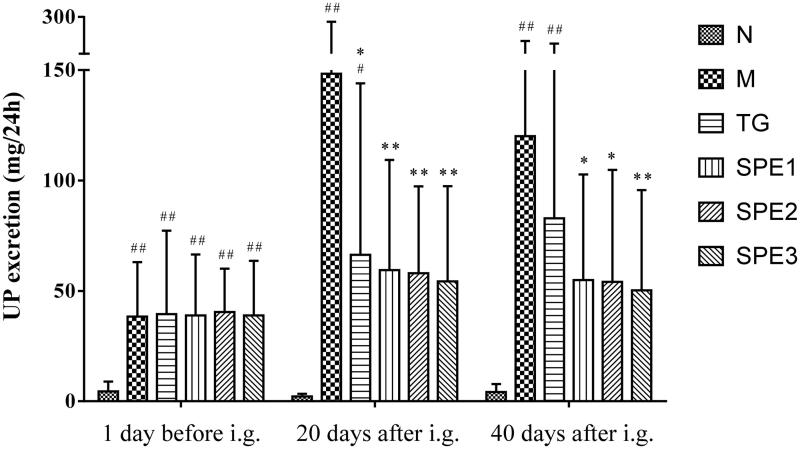
The effect of SPE administration on UP excretion. N: normal control group; M: model group; TG: control medicine group (15 mg/kg TG i.g.); SPE1: low dose treatment group (50 mg/kg SPE i.g.); SPE2: medium dose treatment group (100 mg/kg SPE i.g.); SPE3: high dose treatment group (200 mg/kg SPE i.g.). #*p* < 0.05, ##*p* < 0.01, compared with N group; *p < 0.05, **p < 0.01, compared with M group.

On day 20 after administration of TG and SPE, UP excretion in TG group (66.3 ± 77.7 mg/24 h), and three SPE groups (59.4 ± 49.9, 58.0 ± 39.3, 54.3 ± 43.1 mg/24 h) in particular, was significantly lower than that in M group (148.3 ± 132.3 mg/24 h) (all *p* < 0.01). UP excretion kept on descending on day 40 after SPE administration in all three SPE dose groups (54.9 ± 47.8, 54.1 ± 50.7, 50.2 ± 45.5 mg/24 h) in a positive dose-effect relationship, and was significantly lower than that in M group (120.1 ± 82.1 mg/24 h). The differences in the value of UP excretion between N, M, TG, SPE1, SPE2 and SPE3 groups are shown in Figure 2.

### The effect of SPE on KWMC

**Figure 3.**
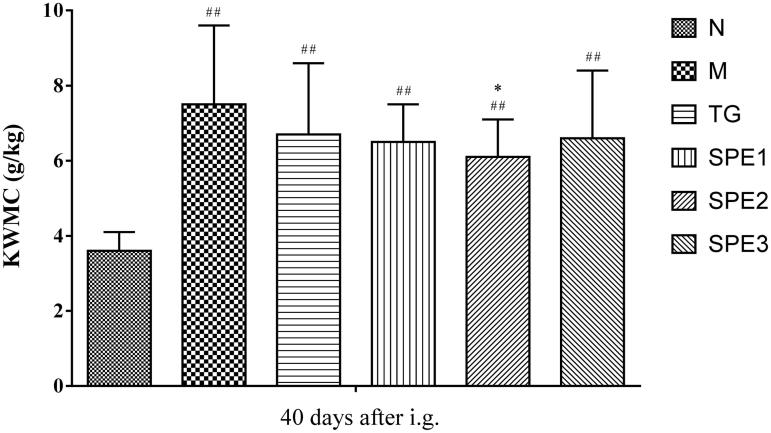
The effect of SPE administration on KWMC. N: normal control group; M: model group; TG: control medicine group (15 mg/kg TG i.g.); SPE1: low dose treatment group (50 mg/kg SPE i.g.); SPE2: medium dose treatment group (100 mg/kg SPE i.g.); SPE3: high dose treatment group (200 mg/kg SPE i.g.). ##*p* < 0.01, compared with N group; *p < 0.05, compared with M group.

As shown in Figure 3, there was a significant difference of KWMC in between SPE2 group and M group (6.1 ± 1.0 g/kg versus 7.5 ± 2.1 g/kg; *p* < 0.05) after 40 days treatment. KWMC in SPE1 and SPE3 groups (6.5 ± 1.0 and 6.6 ± 1.8 g/kg) was also lower than that in M group, while the difference was insignificant compared with M group.

### The effect of SPE on TSP, SA, SC and SUN

As shown in Figure 4, the levels of TSP, SA, SC and SUN were ameliorated in all three SPE groups.

Figure 4.The effect of SPE administration on TSP (a), SA (b), SC (c) and SUN (d). N: normal control group; M: model group; TG: control medicine group (15 mg/kg TG i.g.); SPE1: low dose treatment group (50 mg/kg SPE i.g.); SPE2: medium dose treatment group (100 mg/kg SPE i.g.); SPE3: high dose treatment group (200 mg/kg SPE i.g.). #*p* < 0.05, ##*p* < 0.01, compared with N group; *p < 0.05, **p < 0.01, compared with M group.

**Figure:**
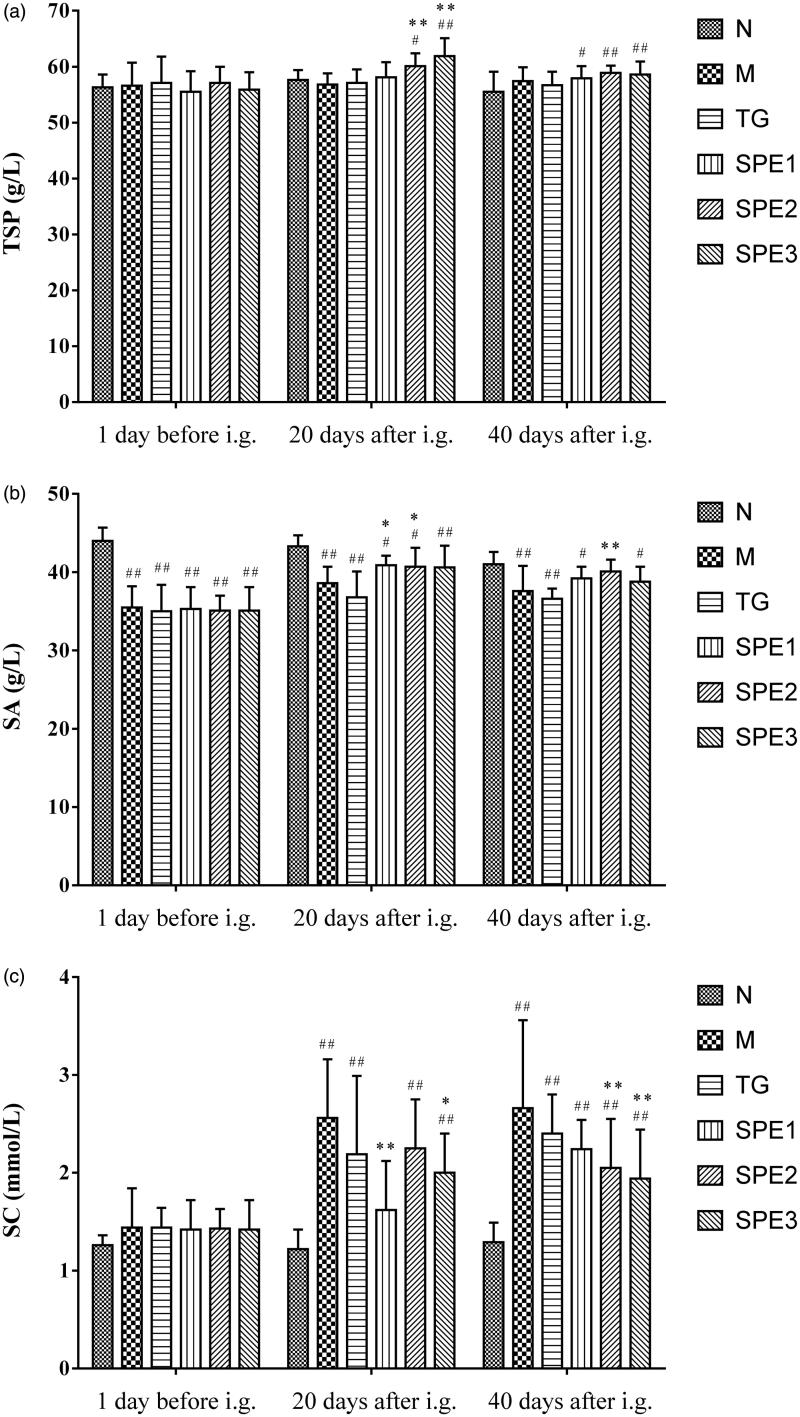

**Figure:**
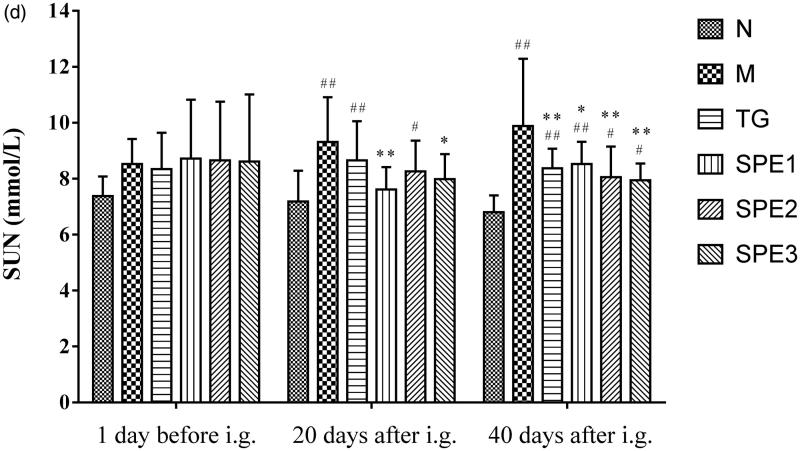

The level of TSP in SPE2 and SPE3 groups rose significantly after 20 days SPE treatment as compared with that in M group (60.1 ± 2.3 and 61.9 ± 3.2 g/L versus 56.8 ± 2.0 g/L; both *p* < 0.01). Compared with M group, TSP was increased in all three SPE groups after 40 days administration (57.9 ± 2.2, 58.9 ± 1.3 and 58.6 ± 2.3 g/L versus 57.4 ± 2.5 g/L) (Figure 4(a)). The level of SA was increased in all three SPE groups after 20 days treatment as compared with that in M group (40.9 ± 1.2, 40.7 ± 2.4 and 40.6 ± 2.8 g/L versus 38.6 ± 2.1 g/L). This effect remained almost the same after 40 days treatment (39.2 ± 1.5, 40.1 ± 1.5 and 38.8 ± 1.9 g/L versus 37.6 ± 3.2 g/L), and was more pronounced in SPE2 group (*p* < 0.01) (Figure 4(b)).

Both SC and SUN levels were decreased in all three SPE groups after 20 days treatment, and this effect was more pronounced in SPE1 and SPE3 groups as compared with that in M group (1.62 ± 0.5, 2.00 ± 0.4 mmol/L versus 2.56 ± 0.6 mmol/L for SC; 7.61 ± 0.8, 7.98 ± 0.9 mmol/L versus 9.31 ± 1.6 mmol/L for SUN; all *p* < 0.05). Compared with M group, both SC and SUN levels continued to decrease in the three SPE groups after 40 days treatment (2.24 ± 0.3, 2.05 ± 0.50 and 1.94 ± 0.5 mmol/L versus 2.66 ± 0.9 mmol/L for SC; 8.52 ± 0.8, 8.05 ± 1.1 and 7.94 ± 0.6 mmol/L versus 9.88 ± 2.4 mmol/L for SUN), especially in SPE2 and SPE3 groups (both *p* < 0.01) (Figure 4(c,d)).

### Histopathological findings

**Figure 5.**
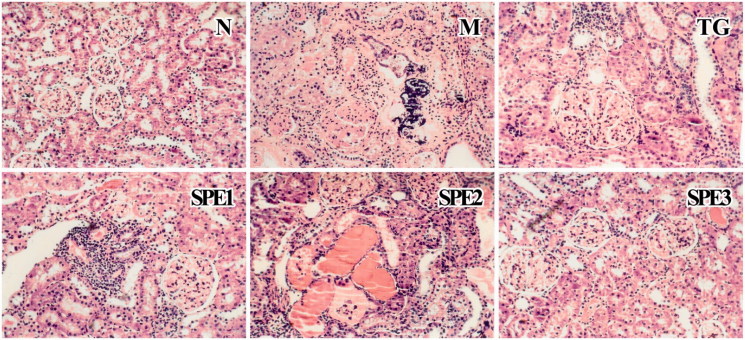
Light micrographic changes of the kidney specimens (stained with HE, 10 × 20). N: normal control group; M: model group; TG: control medicine group (15 mg/kg TG i.g.); SPE1: low dose treatment group (50 mg/kg SPE i.g.); SPE2: medium dose treatment group (100 mg/kg SPE i.g.); SPE3: high dose treatment group (200 mg/kg SPE i.g.).

Light microscopic examination of the renal tissues showed severe glomerular engorgement, apomorphosis of the renal tubular epithelium, interstitial inflammatory infiltration and hypercellularity in all groups except N group. Severe glomerular fibrosis, necrosis and calcification of the renal tubules were observed in M group. Compared with M group, glomerular engorgement and apomorphosis of the renal tubular epithelium were ameliorated significantly in TG, SPE1, SPE2 and SPE3 groups after 40 days treatment. In addition, renal inflammatory infiltration and intumesce were attenuated in all three SPE groups, which were consistent with effect of SPE on KWMC. The changes are shown in Figure 5.

## Discussion

It is common knowledge that human nephritis is mainly due to glomerular deposition and/or immune complex formation (Kasap et al. 2008). These immune complexes, which act as a trigger to deliver cytokines and locally yield more transforming growth factor-β (TGF-β) and platelet-derived growth factor, lead to mesangial cell proliferation and glomerulosclerosis (Isaka et al. 1993; Gómez-Guerrero et al. 2000). MsPGN is one of the most common pathological types of chronic glomerulonephritis in Chinese populations (Li 1989; Cheng et al. 2009). It is defined as a glomerulonephritis with an essentially uniform increase in mesangial cells in all or nearly all glomeruli, of which the specific biopsy findings are diffuse or present focal increase in glomerular mesangial cells and matrix with or without mesangial deposits of immunoglobulins and/or complement (Kasap et al. 2008). Generally, deposition of immune complexes in the mesangial area stimulates mesangial cell proliferation and leads to MsPGN.

Proteinuria is the most common clinical manifestation of kidney disease and the most marked signal of renal damage (Dai et al. 2015). Urine protein excretion level shows an intimate connection with the renal function. Most MsPGN cases are associated with persistent proteinuria or with nephrotic syndrome (Danilewicz and Wągrowska-Danilewicz 2001). Nephrotic syndrome is characterized by proteinuria, hypercholesterolaemia, hypoalbuminaemia and oedema (Maxie and Newman 2007). So, the reduction of proteinuria and the amelioration of blood biochemical indexes are two main targets in assessing the therapeutic efficacy of traditional Chinese medicines.

*Tripterygium wilfordii* Hook. f. (Celastraceae) is a shrub plant. TG is an extract from this plant and has been utilized for 30 years in the treatment of various immune and inflammatory diseases in China, especially rheumatoid arthritis, glomerulonephritis and proliferative glomerulonephritis (Wan et al. 2011). Recent studies have demonstrated the remarkable therapeutic efficacy of TG on proteinuria. But as an immunosuppressive agent, the toxicity and adverse effects of TG are also obvious (Li and Liu 2003; Wan et al. 2011), which limit the more extensive use of TG in clinical practice.

In the present study, we demonstrated the therapeutic efficacy of SPE *in vivo* in improving urinary protein excretion in a rat ICG model, knowing that this nominal model mimics human MsPGN. The anti-proteinuria effect of SPE in rats was observed on day 20 after SPE administration. The efficacy of SPE in decreasing proteinuria was even better than that of TG, especially after 40 days treatment. More importantly, increased TSP and SA values together with decreased SC and SUN values showed that SPE could maintain liver function and renal function effectively. Inflammatory response is the characteristic feature of glomerular disease in immune glomerular injury due to the infiltration of leukocytes and the proliferation of glomerular cells. SPE treatment reduced the KWMC and rectified the histological abnormalities of the kidney. Observably, SPE inhibited inflammatory infiltration of glomerular cells and dropsy of the kidney as shown on light microscopy. Oedema could be treated with diuretic agents such as hydrochlorothiazide in some types of glomerulonephritis (Beitollahi et al. 2015). In addition, as a diuretic agent, SPE is also a possible contributor to reduce oedema and adjust renal function.

The major active components of SPE are rosmarinic acid (content 31.58%) and salvianolic acid B (content 5.52%). Rosmarinic acid, an ester of caffeic acid and 3,4-dihydroxyphenyl lactic acid, is an active polyphenolic phytoconstituent. It has strong anti-oxidative (Lamien-Meda et al. 2010), anti-inflammatory (Parnham and Kesselring 1985; Jiang et al. 2009), anti-allergic (Lee et al. 2008), anti-ultraviolet and anti-radiation activities (Sánchez-Campillo et al. 2009). *In vitro* studies showed that rosmarinic acid inhibited cytokine-induced murine mesangial cell proliferation (Makino et al. 2000). *In vivo* studies showed that oral administration of rosmarinic acid suppressed the proliferation of mesangial cells and glomerular matrix expansion by its antifibrotic effect and anti-oxidative activity on rat MsPGN established by intravenous injection of rabbit anti-rat thymocyte serum to rats (Makino et al. 2002). Salvianolic acid B is a tetramer of caffeic acid, as well as a potent inhibitor of TGF-β1-induced epithelial-mesenchymal transition for the treatment of tubulointerstitial fibrosis (Yao et al. 2009). It showed a strong protective effect on rats with induced diabetic nephropathy (Lee et al. 2003; Kang et al. 2008).

Given the water-soluble polyphenolic compounds in SPE, its efficacy on ICG is closely related to the pharmacological action of rosmarinic acid and salvianolic acid B. The possible pathways are thought to depend on two mechanisms. One is possibly the anti-complementary pathway, knowing that rosmarinic acid possesses the activity to reduce immunohaemolysis by inhibiting the C3-convertase of the classical complement pathway (Englberger et al. 1988). As compounds with polyhydroxylated phenyl rings are highly reactive with the thioester bond in nascent C3b, rosmarinic acid and salvianolic acid B block complement activation by preventing attachment of C3b to the activating surface (Sahu et al. 1999). Improved serum complement level and reduced proteinuria were reported to have a beneficial effect on hypocomplementaemia by inhibiting complement activation in patients with increased serum C3 levels (Fujita et al. 1993). The other possible pathway is the anti-inflammatory and antioxidative pathway. Rosmarinic acid has the inhibitory effect against inflammatory response and the scavenging effect against reactive oxygen radicals (Sánchez-Campillo et al. 2009). It owns an inhibitory activity on the formation of malondialdehyde in human platelets (Gracza et al. 1985). It was reported that malondialdehyde, a kind of lipid peroxidation product, was eliminated by means of suppressing markedly the formation of hydroxyl free radicals and lipid peroxidation reaction by *S. przewalskii* (Cheng and Yang 2003). So, the therapeutic efficacy of SPE may be achieved by the above-mentioned pathways. Furthermore, rosmarinic acid showed very low toxicity with a LD_50_ in mice of 561 mg/kg after intravenous administration (Petersen and Simmonds 2003). So, the safety of utilizing SPE to treat MsPGN is reliable.

## Conclusions

In summary, our present study demonstrated that SPE could reduce proteinuria, regulate protein and lipid metabolisms, attenuate renal inflammatory cell infiltration, and delay the progression of glomerular lesions in a rat ICG model. The results of the present research provide evidences that SPE has a potentiality to become a therapeutic drug for glomerulonephritis. To confirm our conclusion, more studies are required to improve quality control of SPE and clarify its action mechanism against glomerulonephritis for clinical application.
